# Utilizing combusted PET plastic waste and biogenic oils as efficient pour point depressants for crude oil

**DOI:** 10.1038/s41598-024-65563-7

**Published:** 2024-07-10

**Authors:** Mohamed Mohamady Ghobashy, A. M. Rashad, S. K. Attia, A. E. Elsayed, D. I. Osman

**Affiliations:** 1 Radiation Research of Polymer Chemistry Department, National Centre for Radiation Research and Technology (NCRRT), Egyptian Atomic Energy Authority (EAEA), Cairo, Egypt; 2 Evaluation and Analysis Department, Egyptian Petroleum Research Institute, Cairo, Egypt

**Keywords:** Chemical safety, Environmental chemistry, Inorganic chemistry, Materials chemistry, Polymer chemistry, Chemical synthesis

## Abstract

The deposition of paraffin on pipelines during crude oil transit and low-temperature restart processes poses a significant challenge for the oil industry. Addressing this issue necessitates the exploration of innovative materials and methods. Pour point depressants (PPDs) emerge as crucial processing aids to modify paraffin crystallization and enhance crude oil flow. This study focuses on the combustion of polyethylene terephthalate (PET) waste, a prevalent plastic, in two distinct oils (castor and jatropha). The resulting black waxy substances (PET/Castor and PET/Jatropha) were introduced in varying weights (1000, 2000, and 3000 ppm) to crude oil. The PET/castor oil combination demonstrated a remarkable reduction in pour point from 18 to −21 °C at 3000 ppm concentration, significantly more effective than PET/jatropha blends. Substantial decreases in viscosity (up to 75%) and shear stress (up to 72%) were also observed for both blends, most prominently at lower temperatures near the pour point. The synergistic effect of PET and oils as nucleating agents that alter crystallization patterns and restrict crystal growth contributes to this enhanced low-temperature flow. This highlights the potential of PET plastic waste as an economical, abundant, and eco-friendly additive to develop high-performance PPDs for crude oil.

## Introduction

The ubiquitous utilization of crude oil as a vital energy source and feedstock material in modern society is accompanied by a diverse array of technical challenges in its effective processing and transportation. A key impediment confronting the oil industry is the deposition of paraffin wax within oil pipelines and equipment during crude oil transit, especially in regions with cold climates^1^. The crystallization and aggregation of paraffin waxes from crude oil during low-temperature operations results in increased viscosity and yield stress, severely hampering crude oil flowability and posing substantial operational risks and economic losses. Addressing paraffin deposition necessitates the development of innovative solutions to enhance the cold flow properties of crude oil^2^.

Pour point depressants (PPDs) have emerged as a crucial class of processing aids to counter the threats posed by paraffin wax crystallization in crude oil systems. PPDs function by kinetically modifying the crystallization mechanism of wax molecules and restricting further crystal growth. This inhibits the formation of an interconnected paraffin crystal network structure and lowers the pour point temperature, viscosity, and yield stress of crude oil^3^. The oil industry has conventionally employed various polymeric organic-based PPDs including ethylene-vinyl acetate, polyalkylmethacrylates, and polyalkylacrylates to alleviate paraffin-related challenges during crude oil pipeline transportation and restart operations. However, the high costs, limited availability, poor thermal stability, and environmental concerns associated with these traditional PPDs have necessitated the exploration of alternative sustainable materials as efficient PPDs for crude oil^4^.

In this context, recent studies have focused on the utilization of mixtures and composites derived from inexpensive and abundantly available biogenic sources including vegetable oils, animal fat, and lignin as potential PPDs. Vegetable oils display excellent flow and adsorption characteristics owing to the presence of surface-active components like fatty acids and glycerides^5–10^. So, the application of additives derived from vegetable oils for base oil to develop an environmentally green lubricant framework has become important recently^11^. Animal fats and lignin also comprise miscible wax-like hydrocarbons and aromatic compounds capable of disrupting paraffin crystallization^12,13^. While these biogenic materials have shown promise as PPDs, their performance is often limited by availability constraints and high production costs due to the competing food, feed, and material use. Therefore, the quest for alternate sustainable, inexpensive, and widely available materials that can act as PPDs remains an active area of research^14–18^.

An intriguing yet underutilized resource in this regard is plastic waste, especially polyethylene terephthalate (PET), one of the most commonly used plastics globally. PET is employed extensively in packaging applications producing millions of tons of post-consumer waste annually, the majority of which remains unrecycled. Interestingly, the thermal degradation of PET plastic produces an altered black waxy residue resembling the chemical composition of paraffinic wax crystals. This opens the possibility of using such PET combustion products as PPDs by blending them with suitable biogenic oils like castor and jatropha oil. The novel PET-oil blends can potentially act as effective PPDs by disrupting the crystallization pattern and restricting the crystal growth of paraffin wax in crude oil. However, research on the efficacy of such innovative PPD solutions remains limited^19^.

This study aims to investigate the potential of using PET plastic waste combustion products blended with castor and jatropha oils as alternative PPDs for enhancing the low-temperature flow properties of crude oil. The fundamental premise explored is that the altered wax-like PET residue can serve as a nucleating agent modifying the crystallization mechanism of paraffin wax while the oils inhibit crystal aggregation by forming interfacial barriers around the PET-driven crystal surfaces. This can disrupt the 3D paraffin crystal network structure, reducing viscosity and yield stress of crude oil near its pour point temperature. The research focuses on testing this hypothesis by preparing PET-oil blends and evaluating their impact on key rheological indicators including pour point, viscosity, and shear stress of crude oil. The study offers unique insights into an innovative methodology for converting abundantly available plastic waste into value-added PPD products with broad applications in the oil industry. From a sustainability perspective, using unrecycled PET plastic can help alleviate its global environmental impacts while simultaneously addressing the operational hurdles related to paraffin crystallization in crude oil systems. The research highlights the promising synergies between waste plastic utilization and renewable biogenic oils for developing economically viable and eco-friendly solutions to enhance crude oil processability. The fundamental understanding generated can pave the way for further advancements in novel PPD systems derived from sustainable waste resources.

## Materials and methods

### Materials used and sample preparation

The materials used in this study include polyethylene terephthalate (PET) waste, a prevalent plastic, in two distinct oils (castor and jatropha). Toluene (C_7_H_8_), a light crude oil sample, which was received from the Egyptian Petroleum Research Institute (EPRI(. The properties of crude oil were tested following ASTM methods. The physico-chemical characteristics of the crude oils were carried out using ASTM and/or IP standard test methods will be illustrated in the supported file. Table 1 shows the Physicochemical Properties of Crude Oil.

**Table 1:** The physicochemical properties of crude oil.

Experiment	Method	Result
Density @ 15.56 °C	ASTM D-7042	0.7933
Total acid number, mg KOH/ gm	ASTM D-664	0.16
Kinematic viscosity, cSt, @ 50 °C	ASTM D-445	2.41
Total sulfur, wt. %	ASTM D-4294	0.1
Asphaltene content, wt.%	IP-143	0.27
Wax content, wt. %	UOP-64	4.36
Pour point, °C	ASTM D-97	18
Flashpoint, °C	ASTM D-93	−21
Water content, vol. %	ASTM D-4006	0.02
BS & W, vol. %	ASTM D-96	Nil
Ash content, wt. %	ASTM D-482	0.003
Carbon residue, wt. %	ASTM D-189	0.5

### Preparation of black waxy substances (PET/castor and PET/jatropha)

The process of preparing black waxy substances from PET plastic waste, blended with castor oil and jatropha oil, involved several systematic steps. Initially, waste PET plastic (10 g) was meticulously cut into small pieces, and these fragments were mixed thoroughly with (100 ml) of castor oil and jatropha oil separately. The resulting PET-oil mixtures underwent controlled heating on a hot plate, ensuring temperatures remained below 200 °C. During this process, a reaction occurred wherein the PET plastic experienced thermal degradation, transforming into a black waxy substance when blended with the respective oils. The high-temperature treatment induced random scission and crosslinking reactions in the PET polymer chains, yielding a modified black waxy residue reminiscent of paraffinic wax crystals. The resulting PET/Castor and PET/Jatropha blends, obtained from these procedures, were subsequently utilized as pour point depressants for crude oil in further experiments. In summary, the preparation method encompassed cutting PET plastic waste, thoroughly mixing with oils, and controlled heating, resulting in black waxy substances suitable for enhancing crude oil properties.

### Effect of black waxy substances (PET/castor and PET/jatropha) on pour point and viscosity on crude oils

The crude oil samples were warmed up in a water bath to 60 ◦C so that any pre-existing wax was re-dissolved. The samples were doped with the additives using a Hamilton Co. micro-syringe at concentrations of 1000, 2000, and 3000 ppm from PET/Castor and PET/Jatropha blends. The pour point was determined according to the ASTM D 97–93 standard method^20^. The point of pouring waxy crude oil was determined according to ASTM D-97 as follows: A tube was filled with the waxy oil and placed in an oil bath at 50 °C for 30 min, and then the tube was left to cool down to 35 °C. After that, the tube was moved to the pour point device, where the device was set at a temperature less than the pour point of the waxy oil by about 8 °C. The oil’s flowability was monitored by suspending the tube tilted for a period of 5 s, and the temperature at which the oil lost flowability was recorded. Experiments were repeated three times to verify the pour point. The study aimed to evaluate how different concentrations of the PET/Castor and PET/Jatropha blends could influence the pour point and viscosity of the crude oils, providing insights into their potential as additives for improving the low-temperature flow properties and fluidity of waxy crude oils.

### Study of rheological properties

The influence of PPD digitization on oil viscosity was investigated using a Brookfield DV-III ULTRA cone plate viscometer. The oil sample, initially heated to 60 °C, was doped with additives and then transferred to the viscometer cone. Typically, wax-containing crudes are equilibrated for about 1–2 h to fully solubilize all wax and erase potential wax ‘history’ before measurements^21–25^. Shear stress and viscosity were measured over a range of shear rates (2 to 23 rpm, corresponding to 15–172.5 s^−1^) at temperatures of 12, 25, and 40 °C. This analysis aimed to examine how PPD-digitization affects the rheological properties of the oil under different shear rates and temperatures. Detailed information about the prepared samples is provided in Table 1, offering insights into the composition and characteristics of the tested oil samples with PPD-digitization additives.

### Gas chromatography

Paraffin carbon number distribution analysis was conducted using gas chromatography (GC) following ASTM D3328 standards on an Agilent 7890A gas chromatograph. For each analysis, 1 μl of the sample was auto-injected into the system with an inlet temperature and pressure set at 250 °C and 18.54 psi, respectively. Helium served as the carrier gas at a flow rate of 0.455 ml/min. The sample traveled through a 50 m capillary column with an internal diameter of 0.2 and 0.5 μm-thick film, maintaining a maximum temperature of 325 °C. Eluates were detected using a flame ionization detector (FID) also maintained at the column temperature. This analytical approach allowed for the precise determination of the carbon number distribution of paraffins in the samples, providing valuable insights into their composition and characteristics^20^.

### Photomicrographic analysis

Photomicrographic analysis was performed using Leica Microsystems CMS GmbH Emst-Leitz-Str, LEICA DM4 M, Germany, equipped with a Cannon 12 × zoom camera and Leica Qwil software. The crude samples, both neat and treated with additives, were intentionally cooled to temperatures near their pour points and placed on a glass slide covered by a coverslip. The oil samples were heat-treated at 60 °C for 20 min, then loaded onto the microscope hot stage and cooled down from 60 to 15 °C at a cooling rate of 0.5 °C/min, and polarized micrographs of the oil samples at 15 °C were taken. The sample was controlled at 15 °C to keep the growth of the wax crystals. During the whole test, the morphology of the wax crystals was automatically recorded^21^. The objective of the analysis was to capture the alterations in the size and shapes of wax crystals within the samples. Photomicrographs were taken at a magnification of 50× , providing detailed visual information on the morphological changes induced by the additives in the wax crystals present in the crude samples.

## Results and discussion

### FTIR analysis

**Figure 1:**
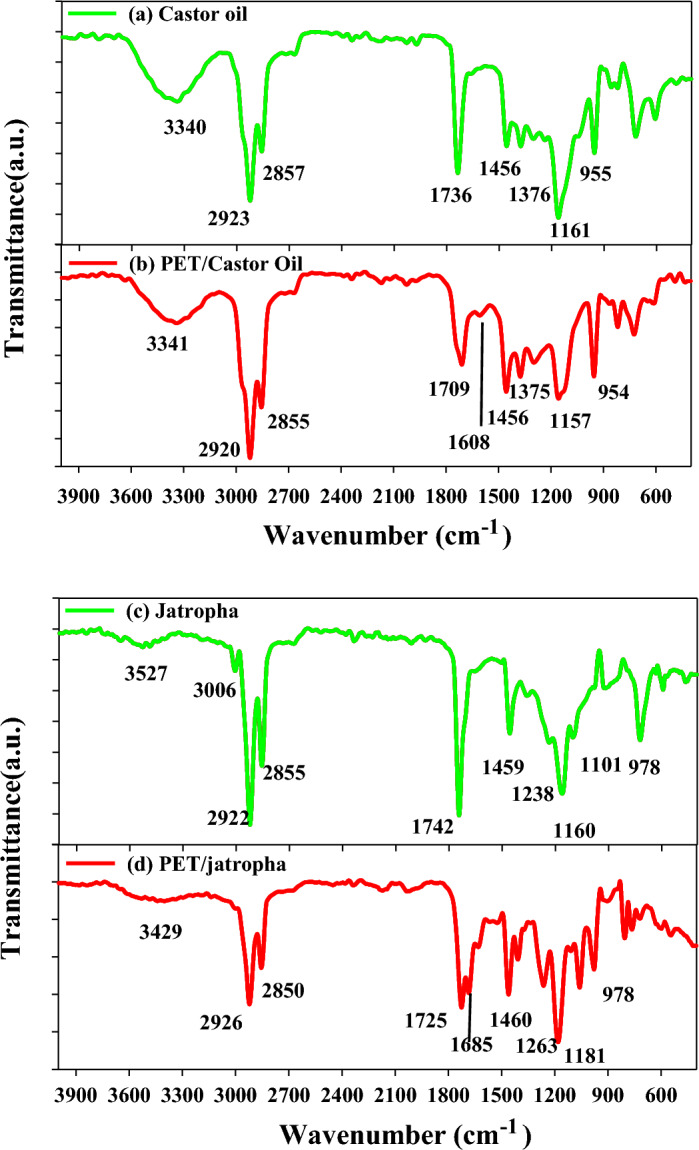
FTIR spectra of (**a**) Castor oil, (**b**) PET/castor oil blend, (**c**) Jatropha oil, and (**d**) PET/Jatropha oil blend.

In the Fourier Transform Infrared (FTIR) analysis, specific peaks in the spectra provide valuable information about the functional groups present in the samples. In the FTIR spectra of Castor oil in Fig. 1a, the peak at 3340 cm^−1^ corresponds to the stretching vibration of O–H groups, indicating the presence of hydroxyl groups in the oil. Peaks at 2923 and 2857 cm^−1^ are associated with C-H stretching vibrations in aliphatic hydrocarbons, characteristic of the oil's lipid content. The peak at 1736 cm^−1^ indicates the presence of carbonyl groups in the form of esters, typical of triglycerides in Castor oil. In contrast, the FTIR peaks for polyethylene terephthalate (PET) blended with Castor oil in Fig. 1b show additional peaks at 3341 cm^−1^, indicative of O–H stretching in PET. The peaks at 2920 and 2855 cm^−1^ suggest the presence of aliphatic C-H groups in PET. Similar functional groups are observed in the FTIR spectra of jatropha oil in Fig. 1c, with characteristic peaks at 3527 cm^-1^ (O–H stretching), 3006 cm^-1^ (C–H stretching), and 2922 cm^−1^ (C–H stretching). The FTIR peaks of PET blended with jatropha oil in Fig. 1d exhibit analogous functional groups but with slight shifts, emphasizing the compatibility of PET with both Castor and jatropha oils. The variations in peak intensities and positions provide insights into the molecular interactions and compatibility of PET with the respective oils. This detailed FTIR analysis enhances our understanding of the chemical composition and compatibility of blended systems, essential for assessing the potential applications of these blends in various industries, including biofuel and polymer engineering^5,26^.

### Effectiveness as pour point depressants

**Figure 2:**
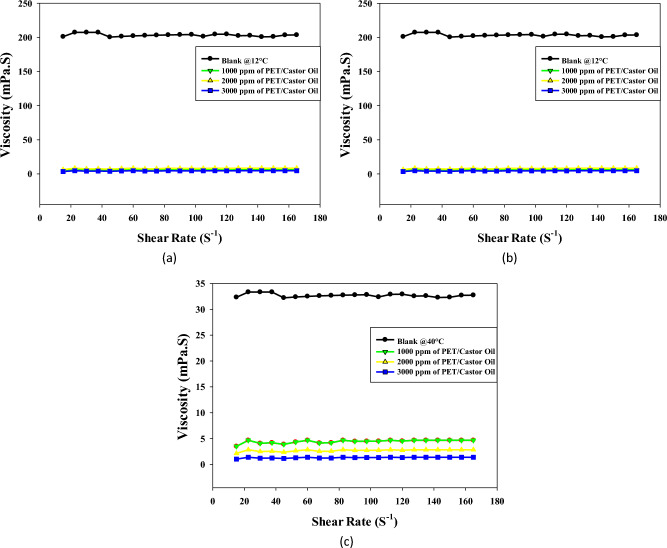
Viscosity of untreated and treated crude oil using PET/castor oil at different temperatures (**a**) 12 °C, (**b**)25 °C, (**c**) 40 °C and different concentrations (1000, 2000 and 3000 ppm).

The data is summarized in Table 2 showing the analysis of pour point temperatures for crude oil treated with varying concentrations (1000, 2000, and 3000 ppm) of PET/castor oil and PET/jatropha oil blends. In Table 2, the analysis at 1000 ppm concentration shows modest reductions in pour point for both PET/castor and PET/jatropha blends compared to untreated crude oil. However, as seen in Table 2, at 2000 ppm concentration, the PET/castor blend achieves a more substantial 30 °C reduction in pour point from 18 to −12 °C, while the PET/jatropha blend lowers it to 0 °C. Figure 2c demonstrates the most dramatic reductions at the highest 3000 ppm concentration. The PET/castor oil blend attains a remarkable 39 °C decrease in pour point from 18 to −21 °C. The PET/jatropha blend also exhibits a notable 27 °C reduction, bringing the pour point down to 9 °C. These observations across Table 2 indicate a clear dose-dependent trend, with higher concentrations of the PET/oil blends resulting in greater pour point depression. This dose dependency underscores the ability to fine-tune the performance of these blends as pour point depressants by adjusting their concentration in crude oil.

**Table 2:** Pour point of treated crude oil with pure castor, jatropha oil, PET/jatropha oil and PET/castor oil.

Samples	Pour point temperatures (^o^C) at different concentrations (ppm)		
	1000	2000	3000
Castor oil	0	−3	−3
PET + castor oil	−9	−12	−21
Jatropha oil	12	6	6
PET + jatropha oil	9	0	−12

Furthermore, the superior performance of the PET/castor oil blend compared to PET/jatropha oil, particularly evident in Table 2, suggests that the choice of biogenic oil also plays a crucial role in the efficacy of these blends. Castor oil, with its unique chemical composition and higher surface-active components, appears to be more effective in synergizing with PET to disrupt paraffin crystallization and improve the low-temperature flow properties of crude oil. The analysis across Table 2 provides compelling evidence for the efficacy of PET/castor oil and PET/jatropha oil blends as novel and sustainable pour point depressants for crude oil. The scientific analysis highlights the complex interplay between PET as a nucleating agent and biogenic oils as crystal growth inhibitors, offering insights into the mechanisms underlying the observed reductions in pour point and opening avenues for further optimization and development of these innovative pour point depressant systems. In addition, Table 2 highlights the significant reduction in pour point temperatures achieved by the addition of PET/castor oil and PET/jatropha oil blends to crude oil. The untreated crude oil exhibits a relatively high pour point of 18 °C, indicating the presence of substantial paraffin wax crystallization that impedes the flow at lower temperatures. The addition of pure castor oil and jatropha oil (without PET) resulted in modest improvements, reducing the pour point to 0 and 12 °C, respectively. This suggests that the biogenic oils, owing to their surface-active components like fatty acids and glycerides, can partially disrupt the wax crystal network and improve the low-temperature flow properties of crude oil. However, the most substantial reductions in pour points were observed when PET was blended with these oils. The PET/castor oil blend achieved a remarkable 39 °C decrease in pour point, from 18 to −21 °C, at the highest concentration of 3000 ppm.

Similarly, the PET/jatropha oil blend lowered the pour point to −12 °C at 3000 ppm, a notable 30 °C reduction from the untreated crude oil. These observations suggest that the PET component in the blends plays a crucial role as a nucleating agent, influencing the crystallization pattern of paraffin wax molecules in crude oil. The PET residue, with its altered chemical composition resembling paraffin waxes, likely acts as a template for wax crystal formation, inducing a change in the crystal morphology and restricting further growth. In addition, our prepared samples give the best pour point depressants and are close to the performance of synthetic oils although our samples are prepared from waste PET recycling, so in the economic and environmental aspects. Our work provides good sustainability as compared with the results obtained from^11,27,28^.

### Rheological properties of the prepared additives on crude oil

Figures 2a–c present a comprehensive analysis of the viscosity profiles of both untreated and PET/castor oil-treated crude oil samples at three distinct temperatures: 12, 25, and 40 °C. The viscosity behavior at different shear rates is pivotal for understanding the flow characteristics of crude oil, especially concerning transportation in pipelines. A key observation from the data is the substantial reduction in crude oil viscosity with the addition of increasing concentrations of the PET/castor oil blend. This effect is consistently evident across all three temperatures, underscoring the versatility of the blend in modifying the rheological properties of crude oil. At each temperature, the viscosity curves of the treated oils systematically shift to lower values compared to the untreated sample at equivalent shear rates. The most significant viscosity reduction is observed for the highest blend concentration of 3000 ppm, signifying the dose-dependent impact of the PET/castor oil blend on viscosity. For instance, at 12 °C and a shear rate of 105 s^−1^, the viscosity drops from 201.11 mPas for the untreated sample to 3.34 mPas for the 3000 ppm treated oil, reflecting an impressive 98.26% decrease. This trend holds true across various shear rates and temperatures, affirming the efficacy of the PET/castor oil blend in lowering crude oil viscosity. An intriguing aspect of the study is the temperature dependency of the viscosity reduction. The viscosity-lowering effect of the PET/castor oil blend is more pronounced at lower temperatures, such as 12 °C, where the blend reduces viscosity by 98.26% compared to untreated crude oil as noted in the study by Eke et al^2^. As the temperature increases to 25 and 40 °C, the reduction in viscosity diminishes, reaching 95% and 96%, respectively. This observation highlights the blend's particular effectiveness in enhancing crude oil flow at colder temperatures, a critical factor for pipelines operating in regions with low ambient temperatures. The non-Newtonian, a slight shear thickening over the shear rate range 10–80 observed in both untreated and treated oils is another noteworthy aspect. The viscosity increases rapidly with increasing shear rate, followed by a more gradual decline at higher shear rates (Table S7 to Table S9). This pseudo-plasticity is crucial for facilitating the flow of crude oil in pipelines. The addition of the PET/castor oil blend preserves this shear-thickening behavior, albeit with a lower degree of nonlinearity, further emphasizing its suitability for pipeline transportation. The substantial viscosity reductions as compared with the untreated sample, especially noticeable at lower temperatures, can be attributed to the pour point depressing ability of the PET/castor oil blend. The PET component likely acts as a nucleating agent, influencing the crystallization pattern of paraffin wax in crude oil. Simultaneously, the castor oil forms an interfacial film around the modified wax crystal surfaces, inhibiting further growth and aggregation. This disruption of the 3D network structure significantly lowers the viscosity and yield stress of the crude oil, validating the blend's potential as an effective and sustainable solution for improving the flow characteristics of crude oils during pipeline transportation. The PET/castor oil blend acts as a pour point depressant, modifying the crystallization behavior of paraffin wax and inhibiting crystal growth and aggregation. This results in a notable reduction in viscosity, particularly at lower temperatures where the viscosity reduction is most prominent. By enhancing the flow properties of crude oil, the blend offers a promising solution for addressing the challenges posed by paraffin deposition and low-temperature restart processes in the oil industry. Its effectiveness in lowering viscosity and improving flow underscores its potential as a reliable and continued pour point depressant.

**Figure 3:**
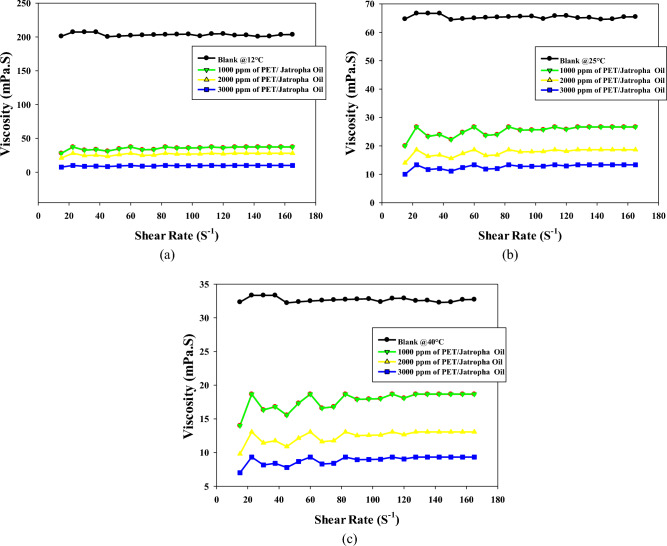
Viscosity of untreated and treated crude oil using PET/jatropha oil at different temperatures (12, 25, 40) ^o^C and different concentrations of PET/jatropha oil ((**a**)1000 ppm and **(b**) 2000 ppm and (**c**) 3000 ppm).

Figures 3a–c illuminate the intricate viscosity profiles of untreated crude oil and the treated with a different concentration of PET/jatropha oil blend across a temperature spectrum of 12, 25, and 40 °C. The introduction of the 3000 ppm PET/jatropha blend exerts a transformative influence, inducing substantial viscosity reductions across all shear rates, with a remarkable 73% reduction at 12 °C and a shear rate of 120 s^−1^. The impact of the PET/jatropha blend on viscosity is most pronounced at 12 °C, where the treated curve diverges markedly from the untreated, and this effect diminishes progressively at elevated temperatures of 25 and 40 °C. Despite the viscosity reductions, the treated jatropha oil maintains its shear thickening over the shear rate range 10–80 s^−1^, Table S4 to Table S6.) substantiated by the continuous decline in the slope of shear stress versus shear rate. Complementing these insights, Fig. 3 provides a quantitative supplement, offering numerical values for the viscosity of crude oil treated with varying doses of PET/jatropha blend at the three temperatures. The trends mirrored in the figures are distinctly evident in Fig. 3, underscoring the maximum reduction in viscosity for the highest 3000 ppm dose, particularly conspicuous at the lower temperature of 12 °C. The substantial viscosity decreases attributed to the pour point depressing action of PET/jatropha blend components find explanation in the altered crystallization pattern induced by PET, acting as a nucleating agent for paraffin crystals, while jatropha oil concurrently forms an interfacial film around crystal surfaces, disrupting the 3D crystal network structure responsible for high viscosity and yield stress. The treated oil, while retaining shear thickening over the shear rate range 10–80 s^−1^, exhibits lower nonlinearity, indicative of a modified rheological profile. The dose and temperature-dependent viscosity reductions observed in Fig. 3 collectively affirm the outstanding potential of PET/jatropha blends promising significant advancements in the realm of crude oil viscosity management for pipeline transportation.

Some key differences can be noted between the two sets of viscosity data. The viscosity reduction caused by the addition of 3000 ppm PET/castor blend to crude oil at 12 °C is in the range of 35–50% compared to untreated crude oil. In contrast, the 3000 ppm PET/jatropha blend leads to a more substantial viscosity decrease of 70–75% in jatropha oil at the same 12 °C temperature. This indicates that the PET/castor blend combination is notably more effective compared to the PET/jatropha. In both oils, the viscosity lowering effect diminishes at higher temperatures, although the reductions remain more significant for jatropha oil treated with PET-jatropha blend. The likely reason is differences in the chemical composition and wax content of the two oils, with jatropha oil containing higher levels of n-paraffins that contribute to crystallization. The comparisons highlight that the performance of PET-oil blends depends on oil type and temperature, with the PET/jatropha system showing superior viscosity reduction especially prominent at low temperatures near the pour point. The results are parallel with the previous findings^29,30^.

**Figure 4:**
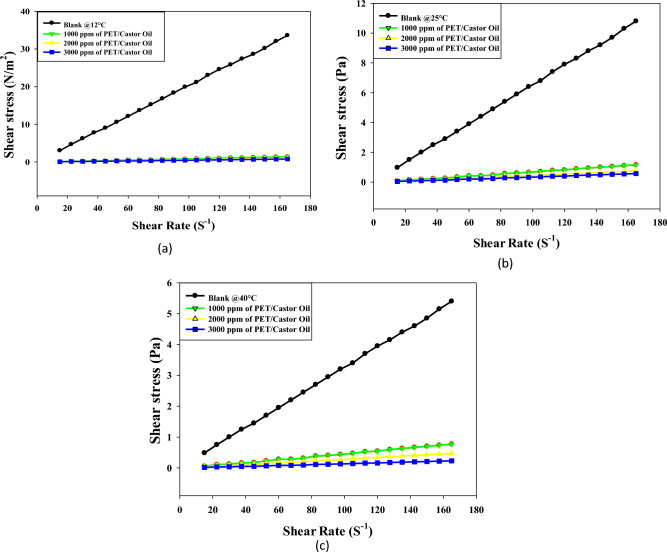
Relation between shear rate and shear stress for untreated and treated crude oil using PET/castor oil in different concentrations (1000, 2000 and 3000 ppm) at different temperatures ((**a**) 12, (**b**) 25, (**c**) 40 °C) ,respectively.

Figures 4a, b, c offer a comprehensive exploration of the shear stress versus shear rate rheograms for crude oil treated with varying doses (1000, 2000, 3000 ppm) of PET/castor oil blend at temperatures of 12, 25, and 40 °C. The observations unveil the intricate rheological behavior of untreated crude oil and the transformative effects induced by the PET/castor blend. The treated crude oil displays shear thickening over the shear rate range 10–80 s^−1^at all three temperatures, with shear stress increasing non-linearly as shear rate escalates, a phenomenon more pronounced at lower temperatures. The addition of PET/castor oil blend engenders dose-dependent reductions in shear stress across the shear rate range, with maximal decreases observed for the 3000 ppm dose. For instance, at 12 °C and a shear rate of 120 s^−1^, shear stress plummets from 24.569 (N/m^2^) for untreated oil to 0.54 (N/m^2^) for the 3000 ppm treated oil with castor blends, representing a substantial 97.80% reduction. The most noteworthy shear stress reductions are observed at 12 °C, diminishing progressively at higher temperatures, highlighting the blend's efficacy, especially near the pour point. The treated oils retain their shear-thinning behavior, albeit with a lower degree of nonlinearity compared to the untreated sample, particularly noticeable at 12 °C. Figure 4 complements these rheograms by providing numerical values that quantify shear stress reductions at discrete shear rates for the three PET/castor blend doses at each temperature. The dose- and temperature-dependent declines in shear stress are clearly evident, reinforcing the transformative impact of the PET/castor blend on crude oil rheology. These pronounced shear stress decreases are attributed to the pour point depressing action of the PET/castor blend, with PET acting as a nucleating agent that alters wax crystallization patterns and castor oil coating the wax crystal surfaces, disrupting the 3D network structure. The substantial reductions in viscosity and shear stress, particularly near the pour point at 12 °C, affirm the outstanding potential of PET/castor blends to improve the low-temperature flow properties of crude oils. This data not only sheds light on the complex rheological changes induced by the blend but also validates its practical application in enhancing the low-temperature flow characteristics of crude oils, thereby contributing to advancements in crude oil transport and processing technologies.

**Figure 5:**
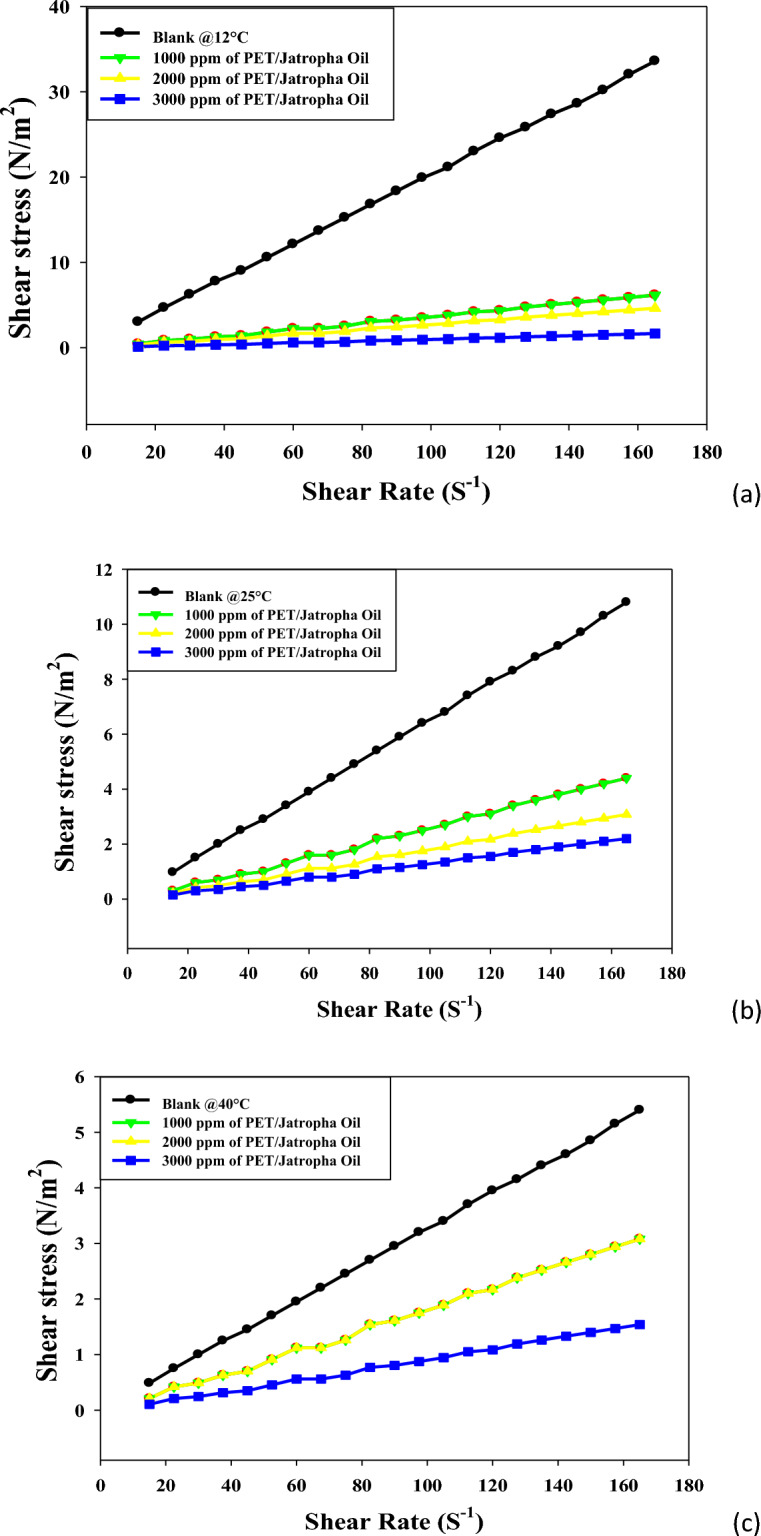
Relation between shear rate and shear stress for untreated and treated crude oil using PET/jatropha oil in different concentrations (1000, 2000 and 3000 ppm) at different temperatures ((**a**) 12, (**b**) 25, (**c**) 40 °C), respectively.

Figures 5a–c elucidate the intricate shear stress versus shear rate rheograms for jatropha oil treated with varying doses (1000, 2000, 3000 ppm) of PET/jatropha blend across temperatures of 12, 25, and 40 °C. The key trends observed unveil the transformative impact of the PET/jatropha blend on the rheological behavior of untreated jatropha oil. The treated crude oil using PET/ jatropha oil in different concentrations exhibits shear thickening behavior, with shear stress increasing nonlinearly as shear rate rises, a behavior more pronounced at lower temperatures. The addition of PET/jatropha blend induces considerable dose-dependent reductions in shear stress across all shear rates, with the most significant decline occurring for the 3000 ppm dose. For example, at 12 °C and a shear rate of 120 s^−1^, shear stress plummets from 24.569 (N/m^2^) for untreated oil to 9.043 (N/m^2^) for the 3000 ppm treated oil, representing a substantial 63% reduction. The most substantial shear stress reductions are observed at 12 °C, diminishing progressively at higher temperatures, with a maximum decrease of 60–70% compared to untreated oil. The treated crude oil using PET/ jatropha oil retain their shear thickening nature but exhibit a lower degree of nonlinearity compared to the untreated sample, especially noticeable at 12 °C^31^. Figure 5 supplements these rheograms by providing numerical values that quantify shear stress decreases at specific shear rates for the three PET/jatropha doses at each temperature, showcasing clear dose- and temperature-dependent trends. The substantial lowering of shear stress induced by the PET/jatropha blend is attributed to its pour point depressing action, akin to the PET/castor blend. PET likely alters the crystallization pattern, while jatropha oil restricts crystal growth by coating modified crystal surfaces, disrupting the 3D network structure. This disruption substantially reduces viscosity and shear stress, most prominently near the pour point at 12 °C. The data robustly verifies the exceptional potential of PET/jatropha blends in improving the low-temperature flow properties of crude oils^32^.

The comprehensive insights from Figs. 5a–c, collectively underscore the superior ability of PET/jatropha blends over the PET/castor system in enhancing the low-temperature flow characteristics of crude oil, presenting promising avenues for advancements in the field of petroleum oil processing and transportation technologies.

Both Figures showcase shear stress versus shear rate rheograms for crude oil and jatropha oil respectively, treated with varying doses of PET blended with castor oil (Fig. 3) and jatropha oil (Fig. 4) at 12, 25 and 40 °C^33^. In both sets of Figures, the untreated oils display shear-thinning behavior with a nonlinear increase of shear stress as the shear rate rises, more pronounced at lower temperatures. The addition of PET-oil blends causes significant dose-dependent reductions in shear stress across all shear rates for both oils. The maximum declines occur with a 3000 ppm blend dose. Figures 4, 5 offer a profound exploration into the rheological transformations induced by PET blends on crude oil and jatropha oil, respectively, shedding light on both commonalities and distinctions. The shared feature of shear-thinning behavior in untreated oils underscores the inherent viscosity reduction observed as shear rates increase, a characteristic often associated with complex fluid dynamics. The dose-dependent reductions in shear stress introduced by PET blends reveal a consistent trend in both figures, emphasizing the potential for tailored pour point depressing solutions to alleviate the viscosity challenges in oil transport, especially at lower temperatures. However, the unique nature of crude oil and jatropha oil introduces noteworthy differences. Crude oil, depicted in Fig. 4, shows a more pronounced shear stress reduction with PET/castor blend treatment, particularly evident at 12 °C. This observation aligns with the historical challenges in transporting crude oil at low temperatures due to increased viscosity.

On the other hand, Fig. 5 illustrates that PET/jatropha blend treatment exhibits a prominent reduction in shear stress for jatropha oil, emphasizing the versatility of such blends for different oil types. The temperature-dependent effects observed in both figures highlight the significance of the pour point depressing action of PET blends, with greater efficacy observed at lower temperatures, where the disruptive influence on wax crystallization and crystal growth is more prominent. The nuanced nature of shear-thinning behavior, especially the differences in nonlinearity observed at lower temperatures, underscores the intricate interactions between the specific oil compositions and the PET blends. These findings collectively advocate for a tailored approach in designing pour point depressing solutions that consider the unique characteristics of different oils, opening avenues for advancements in the field of crude oil processing and transportation technologies. Furthermore, the detailed numerical data presented in Figs. 4, 5, provide quantitative validation to the observed trends and offer a robust foundation for the application of PET blends in optimizing the low-temperature flow properties of diverse oil types.

### Chemical components in crude oil

**Figure 6:**
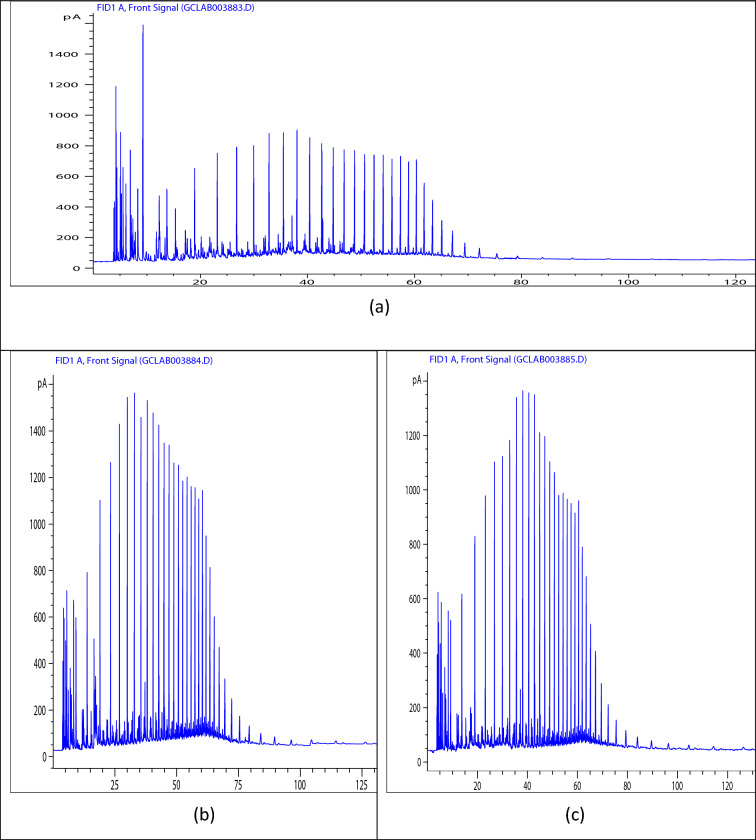
(a) Chromatogram of crude oil (blank). (**b**) Chromatogram of treated crude oil using PET/jatropha oil at concentration 3000 ppm. (**c**) Chromatogram of treated crude oil using PET /castor oil at a concentration 3000 ppm.

The GC analysis presented in Fig. 6 provides critical insights into the chemical compatibility between the crude oil and the PET/oil blends, validating the proposed mechanisms underlying the pour point depressing action of these innovative additives. The detailed understanding gained from the GC data, in conjunction with the rheological analyses, offers a comprehensive perspective on the efficacy of PET/castor oil and PET/jatropha oil blends as sustainable and effective pour point depressants for crude oil. Figure 6 presents the gas chromatography (GC) analysis of the hydrocarbon components present in the crude oil samples—untreated crude oil (blank), crude oil treated with 3000 ppm PET/jatropha oil blend, and crude oil treated with 3000 ppm PET/castor oil blend. Figure 6a shows the chromatogram for the untreated crude oil (blank). The peaks correspond to the distribution of hydrocarbon compounds present, with the x-axis representing retention time (which is related to the carbon number of the hydrocarbons) and the y-axis indicating the peak intensity (which is proportional to the concentration of the respective compounds). The chromatogram reveals that the paraffin carbon number distribution of the blank crude oil has a mode ranging from n-C5 to C41. This broad range indicates the presence of a high concentration of paraffin waxes, which explains the relatively high pour point temperature of 18 °C observed for the untreated crude oil. Figures 6b, c illustrate the chromatograms for crude oil treated with 3000 ppm of PET/jatropha oil and PET/castor oil blends, respectively. The patterns of peaks and their intensities are similar to the untreated crude oil (Fig. 6a), indicating that the overall hydrocarbon composition of the crude oil remains largely unaltered upon treatment with the PET/oil blends. However, a closer analysis of the chromatograms in Figs. 6b, c reveals that the highest percentage of hydrocarbon compounds falls within the range of C10 to C27. Tables (S1, S2, and S3) containing details of the component identified corresponding to each peak is presented in the supported information file. This distribution profile matches the chemical composition of the PET residue and biogenic oils used in the blends, suggesting a high degree of compatibility between the components present in the crude oil and the additives. The compatibility between the chemical components present in the crude oil and the PET/oil blends is a crucial factor contributing to the observed reductions in pour point and improvements in rheological properties, as discussed in the previous sections. The PET residue, with its altered chemical composition resembling paraffin waxes, likely acts as a nucleating agent that modifies the crystallization pattern of the paraffin wax molecules present in the crude oil. Simultaneously, the biogenic oils, which contain surface-active components like fatty acids and glycerides, form interfacial barriers around the modified wax crystal surfaces, further disrupting the formation of an interconnected 3D paraffin network structure. This compatibility between the crude oil components and the PET/oil blends facilitates the synergistic interactions that lead to the substantial reductions in pour point and improvements in flow properties observed in the previous analyses^34^.

### Effect of additives on wax crystal morphology

**Figure 7:**
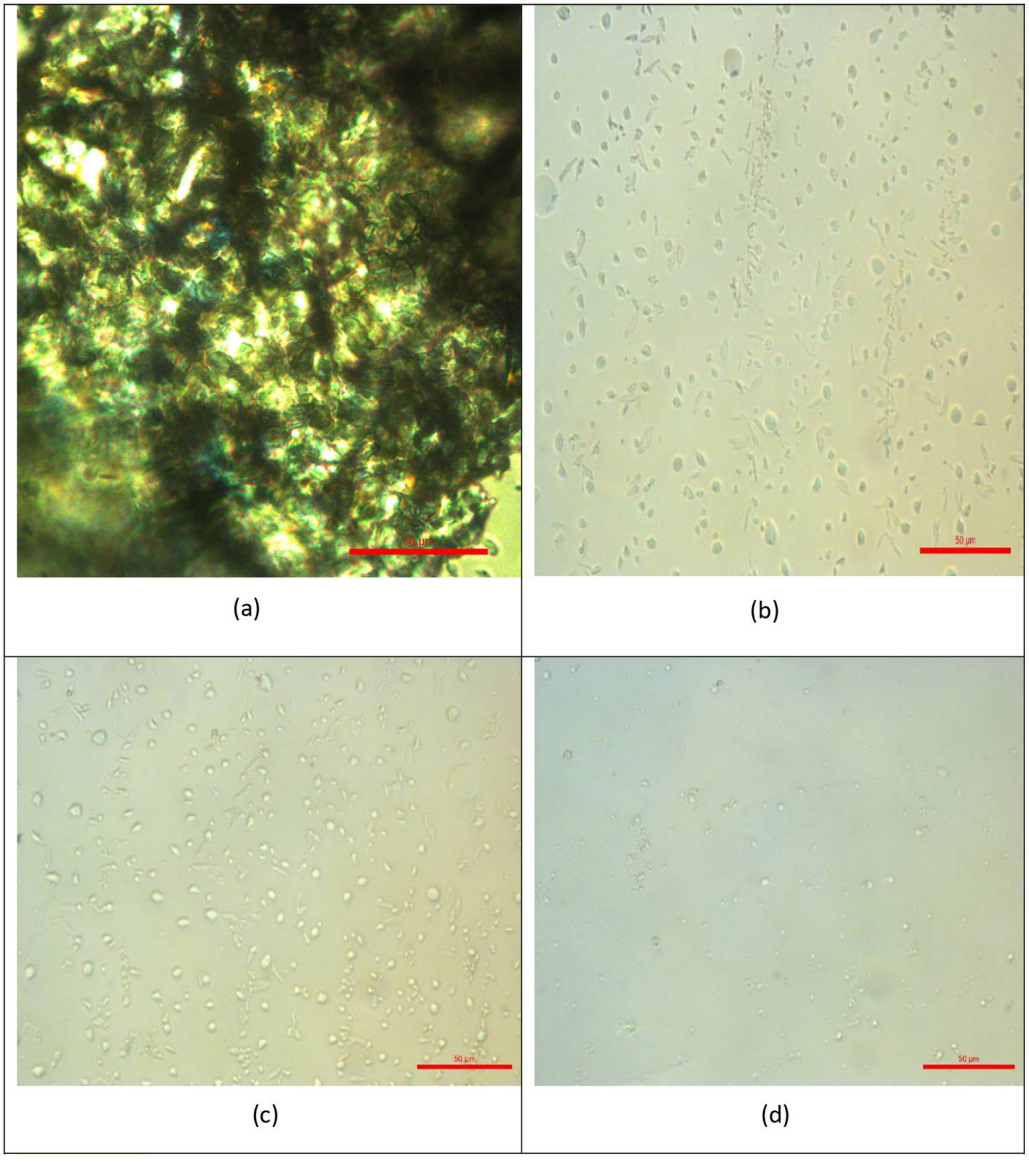
Polarized optical micrograph of untreated crude oil, Treated crude oil using PET /castor oil at different concentration ((**a**) Untreated crude oil 0, (**b**) 1000, (**c**) 2000, and (**d**) 3000 ppm).

Figure 7 presents polarized optical micrographs that illustrate the effect of adding PET/castor oil blends at different concentrations (0, 1000, 2000 ppm, and 3000 ppm) on the microscopic morphology of wax crystals in crude oil. Figure 7a shows the untreated crude oil (0 ppm PET/castor oil), and the wax crystals appear thin, feather-like, and elongated. This morphology indicates that the wax crystals can grow and interconnect to form an extensive network structure, which contributes to the high viscosity and poor flow properties observed in the untreated crude oil, especially at lower temperatures near the pour point. As the concentration of the PET/castor oil blend is increased from 1000 ppm (Fig. 7b) to 2000 ppm (Fig. 7c) and finally to 3000 ppm (Fig. 7d), a remarkable transformation in the shape and distribution of the wax crystals is observed. At 1000 ppm concentration (Fig. 7b), the wax crystals begin to exhibit a more globular and dispersed morphology, indicating that the PET/castor oil blend has started to influence the crystallization process. However, the wax crystals are still relatively large and irregularly distributed. With an increase in concentration to 2000 ppm (Fig. 7c), the wax crystals become more uniform in size and shape, displaying a striped distribution pattern. The outline of the crystals is more well-defined, and the spaces not occupied by wax crystals are clearly increased, suggesting a higher degree of disruption in the interconnected wax crystal network. At the highest concentration of 3000 ppm (Fig. 7d), the wax crystals appear significantly smaller and more evenly dispersed throughout the sample. The outline of the crystals is distinct, and the spaces between them are maximized, indicating a substantial disruption in the formation of an interconnected wax crystal network. Further, with the use of additives, the photomicrographs became much more apparent because of the nonappearance of the accumulated fine crystals and the morphological change in the crystal network structure. The obtained results are parallel with Kamal, R. S, et al.^35^. The changes in the microscopic morphology of the wax crystals, as observed in Fig. 7, can be attributed to the synergistic action of PET and castor oil in the blend. PET, with its altered chemical composition resembling paraffin waxes, likely acts as a nucleating agent, providing additional sites for wax crystal formation and altering the crystallization pattern. Simultaneously, the castor oil forms an interfacial film around the modified wax crystal surfaces, further inhibiting crystal growth and aggregation. This combined effect of PET as a nucleating agent and castor oil as a crystal growth inhibitor leads to the formation of smaller, more uniformly dispersed wax crystals, as seen in Fig. 7d. This disruption of the interconnected wax crystal network is responsible for the substantial reductions in viscosity, shear stress, and pour point observed in the rheological analyses, as discussed in the previous sections. The microscopic analysis presented in Fig. 7 provides direct visual evidence of the transformative impact of PET/castor oil blends on the crystallization behavior of wax in crude oil. The changes observed in crystal morphology and distribution serve as a validation of the proposed mechanisms underlying the pour point depressing action of these innovative additives. The insights gained from this microscopic analysis, in conjunction with the rheological data, offer a comprehensive understanding of the efficacy of PET/castor oil blends as sustainable and effective pour point depressants for crude oil^36^.

## Conclusion

This study demonstrates the promising potential of PET plastic waste combustion products blended with castor and jatropha oils as alternative pour point depressants for crude oil. The PET/oil blends induce remarkable reductions in key rheological indicators including pour point, viscosity, and shear stress of crude oil, especially effective at lower temperatures near pour point. The maximum pour point depression from 18 to −21 °C occurs for PET/castor oil blend at 3000 ppm concentration. PET likely acts as a nucleating agent altering the crystallization pattern, while the oils restrict crystal growth by forming interfacial barriers. This disrupts the paraffin wax network structure, substantially enhancing the low-temperature flow properties of crude oil. The substantial viscosity decreases of up to 75% and shear stress reductions of up to 72% validate this pour point depressing ability. The superior performance of PET/castor blend over the PET/jatropha system highlights the role of oil type in PPD efficacy. This research substantiates the synergistic integration of plastic waste and biogenic oils as a sustainable pathway to develop efficient PPDs to address wax deposition challenges in crude oil. Further exploration of different oil blends and PPD hybrids derived from waste/renewable resources can provide a foundation for next-generation crude oil flow improvement solutions.

### Supplementary Information

Supplementary Information.

## Data Availability

All data generated or analyzed during this study available from the corresponding author on request.
