# Supplementary material for: Utilizing combusted PET plastic waste and biogenic oils as efficient pour point depressants for crude oil
Source: Sci Rep. 2024 Jul 10;14:15887. doi: 10.1038/s41598-024-65563-7 (PMC11237070; doi:10.1038/s41598-024-65563-7)
Supplement: Supplementary file 1 — Supplementary Information. [file 41598_2024_65563_MOESM1_ESM.doc]

**Utilizing Combusted PET Plastic Waste and Biogenic Oils as Efficient Pour Point Depressants for Crude Oil**

**Mohamed Mohamady Ghobashy^1^,**[**Rashad, A.M.**](https://www.scopus.com/authid/detail.uri?authorId=35880402500)**^2^,** [**Attia, S.K.**](https://www.scopus.com/authid/detail.uri?authorId=57194473616)**^2^,** [**Elsayed, A.E.**](https://www.scopus.com/authid/detail.uri?authorId=57217865433)**^2^,**[**Osman, D.I.**](https://www.scopus.com/authid/detail.uri?authorId=56035595800)**^2^.**

1-Radiation Research of Polymer Chemistry Department, National Centre for Radiation Research and Technology (NCRRT), Egyptian Atomic Energy Authority (EAEA), Cairo, Egypt.

2-Evaluation and Analysis Department, Egyptian Petroleum Research Institute, Cairo, Egypt.

**2.1 Materials Used and Sample Preparation.**

The physico-chemical characteristics of the crude oils were carried out using ASTM and/or IPstandard test methods , as follows.

- **Density**

The density of the crude oils and their residues was determined according to the ASTM D7042 method. In this method, the test specimen is introduced into the measuring cells which are at a closely controlled and known temperature. The measuring cells consist of a pair of rotating concentric cylinders and an oscillating U-tube.

- **Kinematic viscosity**

The kinematic viscosity of the crude oils and their residues was determined according to the ASTM D 445 glass capillary viscometer method. In this method, the time is measured in seconds for a fixed volume of liquid to flow under gravity through the capillary of a calibrated viscometer under a reproducible driving head and at a closely controlled temperature. The kinematic viscosity is the product of the measured flow time and the calibration constant of the viscometer. The kinematic viscosity of the crude oils was determined at 50 ^o^C,

- **Sulphur content**

This was carried out according to the ASTM D 4294 methods. In this method, the sample is placed in the beam emitted from an x-ray source. The excitation energy may be derived from a radioactive source or from an x-ray tube. The resultant excited characteristics x-radiation is measured, and the accumulated count compared with counts from previously prepared calibration samples to obtain the sulphur concentration in mass percent. Three groups of calibration samples are required to span the concentration range of 0.01 to 5% sulphur.

- **Carbon residue content**

The carbon residue content of the crude oils and their residues was determined according to the ASTM D 189 method. In this method, the sample after being weighed into a special glass bulb having a capillary opening, is placed in a metal furnace maintained at approximately 550 ^o^C. The sample is thus quickly heated to the point at which all volatile matter is evaporated out of the bulb with or without decomposition, while the heavier residue, remaining in the bulb, undergoes cracking and coking reactions. In the latter portion of the heating period, the coke or carbon residue is subjected to further slow decomposition or slight oxidation due to the possibility of breathing air into the bulb. After a specified heating period, the bulb is removed from the bath, cooled in a desiccator, and again weighed. The residue remaining is calculated as a percentage of the original sample, and reported as Ramsbottom carbon residue.

- **Ash content**

The ash content of the crude oils and their residues was determined according to the ASTM D 482 method. In this method, the sample contained in a suitable vessel, is ignited and allowed to burn until only ash and carbon remain. The carbonaceous residue is reduced to an ash by heating in a muffle furnace at 775 ^o^C, cooled, and weighed.

- **Water content**

The water content of the crude oils was determined according to the ASTM D 4006 distillation method. In this method, the sample is heated under reflux conditions with a water-immiscible solvent which co-distils with the water in the sample. Condensed solvent and water are continuously separated in a trap, the water settles in the graduated section of the trap, and the solvent returns to the distillation flask

- **Wax content**

Wax content was determined by the modified UOP 46-64 method without the oil clarification step). 5g of crude oil was dissolved in 50ml followed by solvent evaporation. The residue was dissolved in 100ml petroleum benzine: acetone mixture (3:1). The solution was placed in deep-freeze (-20 ◦C) for 2 h and filtered. The wax precipitate was driedand weighed and the wax content calculated.

- **Asphaltene content**

Asphaltene content of the crude oils was determined according to the IP 143. To determine the asphaltene content (n-C7 insolubles), mixture of crude oil and heptane in a ratio of 1:30 was refluxed for 60 min. The solution was allowed to cool, and then filtered by vacuum using a 0.45μm Millipore® filter paper

### Total Acid Number

The total acid number (TAN) of all previous samples were measured according to ASTM D664 standard test method using potentiometric automatic titrator [KYOTO AT-400] provided with glass and calomel electrode (non-aqueous electrodes). The titrator was programmed with special method in which the titration was adjusted with slow dosing rate of 0.05 N KOH in isopropyl alcohol.

- **Flash point**

The flash point of the crude oils was determined according to the ASTM D93 distillation method. A brass test cup of specified dimensions, filled to the inside mark with test specimen and fitted with a cover of specified dimensions, is heated and the specimen stirred at specified rates.An ignition source is directed into the test cup at regular intervals with simultaneous interruption of the stirring, until a flash is detected

Table ( S1) Castor oil + PET blend with crude oil Composition by GC

| Castor oil + PET blend with crude oil Composition by GC | |
| --- | --- |
| **Components** | **Wt %** |
| C2 | 0.005 |
| C3 | 0.099 |
| C4 | 0.485 |
| C5 | 1.171 |
| C6 | 2.434 |
| C7 | 2.974 |
| C8 | 4.750 |
| C9 | 4.183 |
| C10 | 3.278 |
| C11 | 3.199 |
| C12 | 3.374 |
| C13 | 3.721 |
| C14 | 4.299 |
| C15 | 4.557 |
| C16 | 4.297 |
| C17 | 4.957 |
| C18 | 4.168 |
| C19 | 3.890 |
| C20 | 3.615 |
| C21 | 3.718 |
| C22 | 3.386 |
| C23 | 3.334 |
| C24 | 3.177 |
| C25 | 3.087 |
| C26 | 3.050 |
| C27 | 3.224 |
| C28 | 2.774 |
| C29 | 2.713 |
| C30 | 2.408 |
| C31 | 2.344 |
| C32 | 1.841 |
| C33 | 1.554 |
| C34 | 1.182 |
| C35 | 0.730 |
| C36 | 0.592 |
| C37 | 0.388 |
| C38 | 0.418 |
| C39 | 0.282 |
| C40 | 0.206 |
| C41 | 0.137 |
| **Totals** | **100.000** |

Table ( S2) Blank oil(crude oil) Composition by GC

| Blank Composition | |
| --- | --- |
| Components | Wt % |
| C2 | 0.000 |
| C3 | 0.069 |
| C4 | 0.870 |
| C5 | 2.399 |
| C6 | 4.926 |
| C7 | 6.097 |
| C8 | 11.823 |
| C9 | 6.240 |
| C10 | 5.657 |
| C11 | 3.927 |
| C12 | 3.678 |
| C13 | 4.424 |
| C14 | 4.524 |
| C15 | 5.095 |
| C16 | 3.441 |
| C17 | 3.801 |
| C18 | 2.890 |
| C19 | 2.638 |
| C20 | 2.739 |
| C21 | 2.548 |
| C22 | 2.294 |
| C23 | 2.302 |
| C24 | 2.221 |
| C25 | 2.352 |
| C26 | 2.289 |
| C27 | 2.274 |
| C28 | 1.846 |
| C29 | 1.578 |
| C30 | 1.397 |
| C31 | 1.161 |
| C32 | 0.841 |
| C33 | 0.595 |
| C34 | 0.379 |
| C35 | 0.190 |
| C36 | 0.109 |
| C37 | 0.124 |
| C38 | 0.109 |
| C39 | 0.097 |
| C40 | 0.044 |
| C41 | 0.013 |
| Totals | 100.000 |

Table (S3) Jatropha oil+ PET blend with crude oil Composition by GC

| Jatropha oil+ PET blend with crude oil Composition by GC | |
| --- | --- |
| Components | Wt % |
| C2 | 0.004 |
| C3 | 0.086 |
| C4 | 0.479 |
| C5 | 1.185 |
| C6 | 2.454 |
| C7 | 3.311 |
| C8 | 4.960 |
| C9 | 7.087 |
| C10 | 3.730 |
| C11 | 3.821 |
| C12 | 3.782 |
| C13 | 3.757 |
| C14 | 3.873 |
| C15 | 3.878 |
| C16 | 3.556 |
| C17 | 4.050 |
| C18 | 3.555 |
| C19 | 3.253 |
| C20 | 3.163 |
| C21 | 3.315 |
| C22 | 3.254 |
| C23 | 3.311 |
| C24 | 3.195 |
| C25 | 3.252 |
| C26 | 3.050 |
| C27 | 3.115 |
| C28 | 3.008 |
| C29 | 2.971 |
| C30 | 2.442 |
| C31 | 2.234 |
| C32 | 1.887 |
| C33 | 1.478 |
| C34 | 1.161 |
| C35 | 0.735 |
| C36 | 0.451 |
| C37 | 0.371 |
| C38 | 0.307 |
| C39 | 0.222 |
| C40 | 0.145 |
| C41 | 0.114 |
| Totals | 100.000 |


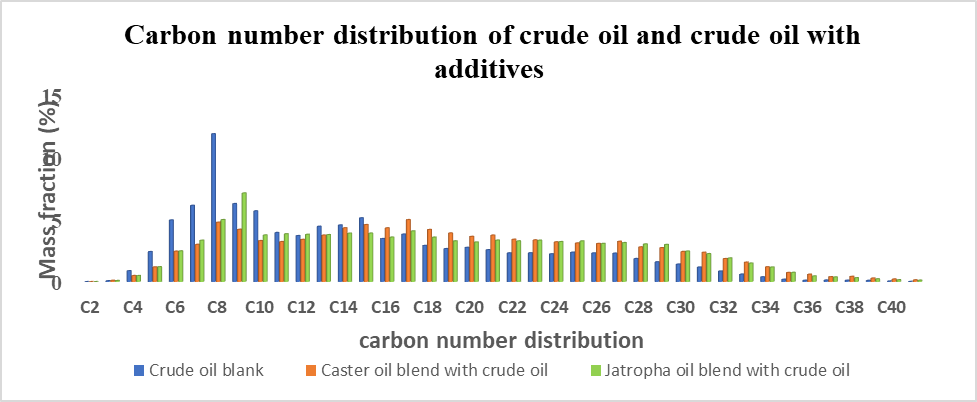


Fig ( S1) Carbon number distribution for different samples

Table (S4) Viscosity Using PET/jatropha oil at Temperature 12^o^C and Different Concentrations of PET/jatropha oil ((a)1000ppm and (b) 2000 ppm and (c) 3000ppm)

| shear rate | Jatropha oil blend | | |
| --- | --- | --- | --- |
|  | Viscosity @ 12 ^o^C | | |
|  | (a)1000 ppm | (b)2000 ppm | ©3000 ppm |
| 15 | 28.00 | 21.00 | 7.50 |
| 22.5 | 37.33 | 28.00 | 10.00 |
| 30 | 32.67 | 24.50 | 8.75 |
| 37.5 | 33.60 | 25.20 | 9.00 |
| 45 | 31.11 | 23.33 | 8.33 |
| 52.5 | 34.67 | 26.00 | 9.29 |
| 60 | 37.33 | 28.00 | 10.00 |
| 67.5 | 33.19 | 24.89 | 8.89 |
| 75 | 33.60 | 25.20 | 9.00 |
| 82.5 | 37.33 | 28.00 | 10.00 |
| 90 | 35.78 | 26.83 | 9.58 |
| 97.5 | 35.90 | 26.92 | 9.62 |
| 105 | 36.00 | 27.00 | 9.64 |
| 112.5 | 37.33 | 28.00 | 10.00 |
| 120 | 36.17 | 27.13 | 9.69 |
| 127.5 | 37.33 | 28.00 | 10.00 |
| 135 | 37.33 | 28.00 | 10.00 |
| 142.5 | 37.33 | 28.00 | 10.00 |
| 150 | 37.33 | 28.00 | 10.00 |
| 157.5 | 37.33 | 28.00 | 10.00 |
| 165 | 37.33 | 28.00 | 10.00 |
| 172.5 | 37.33 | 28.00 | 10.00 |

Table (S5) Viscosity Using PET/jatropha oil at Temperature 25^o^C and Different Concentrations of PET/jatropha oil ((a)1000ppm and (b) 2000 ppm and (c) 3000ppm)

| shear rate | Jatropha oil blend | | |
| --- | --- | --- | --- |
|  | Viscosity @ 25 ^o^C | | |
|  | (a)1000 ppm | (b)2000 ppm | ( c )3000 ppm |
| 15 | 20.00 | 14.00 | 10.00 |
| 22.5 | 26.67 | 18.67 | 13.33 |
| 30 | 23.33 | 16.33 | 11.67 |
| 37.5 | 24.00 | 16.80 | 12.00 |
| 45 | 22.22 | 15.56 | 11.11 |
| 52.5 | 24.76 | 17.33 | 12.38 |
| 60 | 26.67 | 18.67 | 13.33 |
| 67.5 | 23.70 | 16.59 | 11.85 |
| 75 | 24.00 | 16.80 | 12.00 |
| 82.5 | 26.67 | 18.67 | 13.33 |
| 90 | 25.56 | 17.89 | 12.78 |
| 97.5 | 25.64 | 17.95 | 12.82 |
| 105 | 25.71 | 18.00 | 12.86 |
| 112.5 | 26.67 | 18.67 | 13.33 |
| 120 | 25.83 | 18.08 | 12.92 |
| 127.5 | 26.67 | 18.67 | 13.33 |
| 135 | 26.67 | 18.67 | 13.33 |
| 142.5 | 26.67 | 18.67 | 13.33 |
| 150 | 26.67 | 18.67 | 13.33 |
| 157.5 | 26.67 | 18.67 | 13.33 |
| 165 | 26.67 | 18.67 | 13.33 |
| 172.5 | 26.67 | 18.67 | 13.33 |

Table (S6) Viscosity Using PET/jatropha oil at Temperature 40^o^C and Different Concentrations of PET/jatropha oil ((a)1000ppm and (b) 2000 ppm and (c) 3000ppm)

| Shear rate | Jatropha oil blend | | |
| --- | --- | --- | --- |
|  | Viscosity @ 40 ^o^C | | |
|  | ( a) 1000 ppm | ( b) 2000 ppm | ( c )3000 ppm |
| 15 | 14.00 | 9.80 | 7.00 |
| 22.5 | 18.67 | 13.07 | 9.33 |
| 30 | 16.33 | 11.43 | 8.17 |
| 37.5 | 16.80 | 11.76 | 8.40 |
| 45 | 15.56 | 10.89 | 7.78 |
| 52.5 | 17.33 | 12.13 | 8.67 |
| 60 | 18.67 | 13.07 | 9.33 |
| 67.5 | 16.59 | 11.61 | 8.30 |
| 75 | 16.80 | 11.76 | 8.40 |
| 82.5 | 18.67 | 13.07 | 9.33 |
| 90 | 17.89 | 12.52 | 8.94 |
| 97.5 | 17.95 | 12.56 | 8.97 |
| 105 | 18.00 | 12.60 | 9.00 |
| 112.5 | 18.67 | 13.07 | 9.33 |
| 120 | 18.08 | 12.66 | 9.04 |
| 127.5 | 18.67 | 13.07 | 9.33 |
| 135 | 18.67 | 13.07 | 9.33 |
| 142.5 | 18.67 | 13.07 | 9.33 |
| 150 | 18.67 | 13.07 | 9.33 |
| 157.5 | 18.67 | 13.07 | 9.33 |
| 165 | 18.67 | 13.07 | 9.33 |
| 172.5 | 18.67 | 13.07 | 9.33 |

Table (S7) Viscosity Using PET/Castor oil at Temperature 12^o^C and Different Concentrations of PET/jatropha oil ((a)1000ppm and (b) 2000 ppm and (c) 3000ppm)

| Shear rate | Castor oil blend | | |
| --- | --- | --- | --- |
|  | Viscosity @ 12 ^o^C | | |
|  | ( a) 1000 ppm | (b) 2000 ppm | ( c )3000 ppm |
| 15 | 6.30 | 4.54 | 3.48 |
| 22.5 | 8.40 | 6.05 | 4.65 |
| 30 | 7.35 | 5.29 | 4.06 |
| 37.5 | 7.56 | 5.44 | 4.18 |
| 45 | 7.00 | 5.04 | 3.87 |
| 52.5 | 7.80 | 5.62 | 4.31 |
| 60 | 8.40 | 6.05 | 4.65 |
| 67.5 | 7.47 | 5.38 | 4.13 |
| 75 | 7.56 | 5.44 | 4.18 |
| 82.5 | 8.40 | 6.05 | 4.65 |
| 90 | 8.05 | 5.80 | 4.45 |
| 97.5 | 8.08 | 5.82 | 4.47 |
| 105 | 8.10 | 5.83 | 4.48 |
| 112.5 | 8.40 | 6.05 | 4.65 |
| 120 | 8.14 | 5.86 | 4.50 |
| 127.5 | 8.40 | 6.05 | 4.65 |
| 135 | 8.40 | 6.05 | 4.65 |
| 142.5 | 8.40 | 6.05 | 4.65 |
| 150 | 8.40 | 6.05 | 4.65 |
| 157.5 | 8.40 | 6.05 | 4.65 |
| 165 | 8.40 | 6.05 | 4.65 |
| 172.5 | 8.40 | 6.05 | 4.65 |

Table (S8) Viscosity Using PET/Castor oil at Temperature 25^o^C and Different Concentrations of PET/jatropha oil ((a)1000ppm and (b) 2000 ppm and (c) 3000ppm)

| Shear rate | Castor oil blend | | |
| --- | --- | --- | --- |
|  | Viscosity @ 25 ^o^C | | |
|  | ( a) 1000 ppm | (b) 2000 ppm | ( c )3000 ppm |
| 15 | 5.25 | 3.41 | 2.60 |
| 22.5 | 7.00 | 4.55 | 3.47 |
| 30 | 6.13 | 3.98 | 3.03 |
| 37.5 | 6.30 | 4.10 | 3.12 |
| 45 | 5.83 | 3.79 | 2.89 |
| 52.5 | 6.50 | 4.23 | 3.22 |
| 60 | 7.00 | 4.55 | 3.47 |
| 67.5 | 6.22 | 4.04 | 3.08 |
| 75 | 6.30 | 4.10 | 3.12 |
| 82.5 | 7.00 | 4.55 | 3.47 |
| 90 | 6.71 | 4.36 | 3.32 |
| 97.5 | 6.73 | 4.38 | 3.33 |
| 105 | 6.75 | 4.39 | 3.34 |
| 112.5 | 7.00 | 4.55 | 3.47 |
| 120 | 6.78 | 4.41 | 3.36 |
| 127.5 | 7.00 | 4.55 | 3.47 |
| 135 | 7.00 | 4.55 | 3.47 |
| 142.5 | 7.00 | 4.55 | 3.47 |
| 150 | 7.00 | 4.55 | 3.47 |
| 157.5 | 7.00 | 4.55 | 3.47 |
| 165 | 7.00 | 4.55 | 3.47 |
| 172.5 | 7.00 | 4.55 | 3.47 |

Table (S9) Viscosity Using PET/Castor oil at Temperature 40^o^C and Different Concentrations of PET/jatropha oil ((a)1000ppm and (b) 2000 ppm and (c) 3000ppm)

| Shear rate | Castor oil blend | | |
| --- | --- | --- | --- |
|  | Viscosity @ 40 ^o^C | | |
|  | ( a) 1000 ppm | (b) 2000 ppm | ( c)3000 ppm |
| 15 | 3.50 | 2.14 | 1.04 |
| 22.5 | 4.67 | 2.85 | 1.39 |
| 30 | 4.08 | 2.49 | 1.21 |
| 37.5 | 4.20 | 2.56 | 1.25 |
| 45 | 3.89 | 2.37 | 1.16 |
| 52.5 | 4.33 | 2.64 | 1.29 |
| 60 | 4.67 | 2.85 | 1.39 |
| 67.5 | 4.15 | 2.53 | 1.23 |
| 75 | 4.20 | 2.56 | 1.25 |
| 82.5 | 4.67 | 2.85 | 1.39 |
| 90 | 4.47 | 2.73 | 1.33 |
| 97.5 | 4.49 | 2.74 | 1.33 |
| 105 | 4.50 | 2.75 | 1.34 |
| 112.5 | 4.67 | 2.85 | 1.39 |
| 120 | 4.52 | 2.76 | 1.34 |
| 127.5 | 4.67 | 2.85 | 1.39 |
| 135 | 4.67 | 2.85 | 1.39 |
| 142.5 | 4.67 | 2.85 | 1.39 |
| 150 | 4.67 | 2.85 | 1.39 |
| 157.5 | 4.67 | 2.85 | 1.39 |
| 165 | 4.67 | 2.85 | 1.39 |
| 172.5 | 4.67 | 2.85 | 1.39 |
